# Direct Observation Assessment of Milestones: Problems with Reliability

**DOI:** 10.5811/westjem.2015.9.27270

**Published:** 2015-10-22

**Authors:** Meghan Schott, Raashee Kedia, Susan B. Promes, Thomas Swoboda, Kevin O’Rourke, Walter Green, Rachel Liu, Brent Stansfield, Sally A. Santen

**Affiliations:** * University of California, San Francisco, Department of Emergency Medicine, San Francisco, California; † Icahn School of Medicine at Mount Sinai, Department of Emergency Medicine, New York, New York; ‡ Louisiana State University Health Sciences Center – Shreveport, Department of Emergency Medicine, Shreveport, Louisiana; § New York Methodist Hospital, Department of Emergency Medicine, Brooklyn, New York; ¶ Parkland Memorial Hospital, Department of Emergency Medicine, Dallas, Texas; || Yale University, Department of Emergency Medicine, New Haven, Connecticut; # University of Michigan Medical School, Department of Medical Education, Ann Arbor, Michigan

## Abstract

**Introduction:**

Emergency medicine (EM) milestones are used to assess residents’ progress. While some milestone validity evidence exists, there is a lack of standardized tools available to reliably assess residents. Inherent to this is a concern that we may not be truly measuring what we intend to assess. The purpose of this study was to design a direct observation milestone assessment instrument supported by validity and reliability evidence. In addition, such a tool would further lend validity evidence to the EM milestones by demonstrating their accurate measurement.

**Methods:**

This was a multi-center, prospective, observational validity study conducted at eight institutions. The Critical Care Direct Observation Tool (CDOT) was created to assess EM residents during resuscitations. This tool was designed using a modified Delphi method focused on content, response process, and internal structure validity. Paying special attention to content validity, the CDOT was developed by an expert panel, maintaining the use of the EM milestone wording. We built response process and internal consistency by piloting and revising the instrument. Raters were faculty who routinely assess residents on the milestones. A brief training video on utilization of the instrument was completed by all. Raters used the CDOT to assess simulated videos of three residents at different stages of training in a critical care scenario. We measured reliability using Fleiss’ kappa and interclass correlations.

**Results:**

Two versions of the CDOT were used: one used the milestone levels as global rating scales with anchors, and the second reflected a current trend of a checklist response system. Although the raters who used the CDOT routinely rate residents in their practice, they did not score the residents’ performances in the videos comparably, which led to poor reliability. The Fleiss’ kappa of each of the items measured on both versions of the CDOT was near zero.

**Conclusion:**

The validity and reliability of the current EM milestone assessment tools have yet to be determined. This study is a rigorous attempt to collect validity evidence in the development of a direct observation assessment instrument. However, despite strict attention to validity evidence, inter-rater reliability was low. The potential sources of reducible variance include rater- and instrument-based error. Based on this study, there may be concerns for the reliability of other EM milestone assessment tools that are currently in use.

## INTRODUCTION

As the next phase of competency-based assessment, the Accreditation Council for Graduate Medical Education (ACGME) developed milestones through expert consensus and comprehensive literature reviews.^1^,^2^ The milestones are specialty-specific outcome-based expectations used to evaluate physicians’ progress during residency and readiness to complete training, as well as to evaluate residency programs.^3^ The ACGME requires semiannual evaluations on 23 emergency medicine (EM) sub-competencies and over 120 milestones. This competency-based assessment makes fundamental skills explicit for learners and allows attendings to evaluate learners based on specific criteria.

The EM milestones were developed with attention to content validity by using an expert panel, building upon the “EM Model,” and the work of the American Board of EM.^4^ The workgroup that developed the milestones acknowledged that “the next challenge to each residency and the specialty as a whole is the development of objective measures of milestone subcompetency assessment” and the “issues of assessment tool validity and of inter-rater reliability will need to be studied and addressed as various assessment tools are developed, piloted, and put into widespread use.”^4^

We must ensure that we are able to accurately measure what we intend to assess; however, as of yet, there are no reliable assessment tools with clear validity evidence. A research group convened to develop such a tool. Workplace-based direct observation is key to assessing performance, and in the ED an attending physician is routinely present to supervise the initial resuscitation of a critically ill patient. The purpose of this study was to design and validate a tool, the Critical Care Direct Observation Tool (CDOT), which allows for direct observation of EM residents on multiple ACGME milestones during the first several minutes of a critical resuscitation.

## METHODS

### Study Design and Setting

A multi-center, prospective, observational study was conducted at eight academic institutions distributed throughout the country. The study was approved by the review boards at each participating site and was deemed to be exempt as an educational tool without identification of human subjects. This study was part of the Medical Education Research Certification (MERC) program and the Council of Residency Directors (CORD). The research team was composed of five faculty members, two fellows, and one resident from U.S. academic centers.

### Instrument Development

The CDOT’s design was based on the ACGME EM Milestones.^2^ The researchers met via in-person meetings and through monthly conference calls to create the tool using a modified Delphi process.^5^ Consensus was reached that an ideal tool would 1) evaluate multiple milestones in an efficient manner using direct observation, 2) be easy to use and be generalizable, and 3) include reliability and validity evidence. When discussing what clinical scenarios would be optimal for direct observation, the panel determined that resuscitations of unstable patients often requires the resident to demonstrate breadth of knowledge, advanced patient care, team management, and communication skills. Further, faculty are routinely present during resuscitation; hence, assessment would not require additional observation time.

The CDOT was developed and revised in spring of 2013 following the principles of content validity, response process, and internal structure (Table). 6 Evidence of content validity is found in the table, with the use of language from the milestones in order to avoid ambiguity and to improve individual test item quality. We added clarification to several items to align with direct observation assessments.

Validity evidence supporting internal structure and response process are noted in the table. Further, the scoring algorithm was derived directly from the ACGME milestones. We modified the scoring categories to decrease ambiguity and consequentially minimize variability in the final score.^9^ Feasibility and response process were determined by field testing and revision of the instrument.

### Study Protocol

Part one of the study focused on building validity evidence for the CDOT (Table). After the initial CDOT was designed, ensuring content validity for the purpose of response process validity, each of the physician investigators piloted the tool on 29 resident field observations. Feedback was solicited on the performance of the CDOT, and the tool was subsequently revised. Two versions of the CDOT were developed: a Checklist approach (Appendix 1) and a Milestone Rating Scale (Appendix 2), assessing nine of the 23 sub-competencies.

Part two of the study involved evaluating the two versions of the CDOT for inter-rater reliability (internal structure). Faculty reviewed a standardized video of three residents with different levels of training caring for a patient with an aortic dissection and scored them with both CDOTs. The training provided to the faculty assessors consisted of a 10-minute training video introducing the elements of the tool prior to the three resident-patient encounters but did not give specific instruction on how to implement the CDOT. The Checklist format was used with the video review a total of 25 times, and the sub-competency Milestone level CDOT was used a total of 16 times.

### Data Analysis

We coded Checklist responses as categorical variables: “Not Applicable,” “Not Performed,” “Performed Partially,” and “Performed Adequately.” For each checklist item, inter-rater reliability was estimated using Fleiss’ kappa. 7 We compared values of kappa to Landis and Koch’s levels of inter-rater agreement. 8

For the Milestone Rating Scale, respondents scored residents using the standard milestone levels. The “NA” and zero ratings were treated as missing data. We estimated inter-rater reliability using the intra-class correlation type 1 (ICC1), which estimates the percentage of the rating variance attributable to differences in trainee performance level. Higher ICC1 scores indicate more inter-rater agreement with ICC1=0.80 indicating adequate agreement. We performed all analyses in R version 2.11.1 (R Development Core Team, 2010) using the “irr” package version 0.83 for the computation of Fleiss’ kappa and the “multilevel” package version 2.3 for the computation of ICC1.

## RESULTS

**Figure 1:**
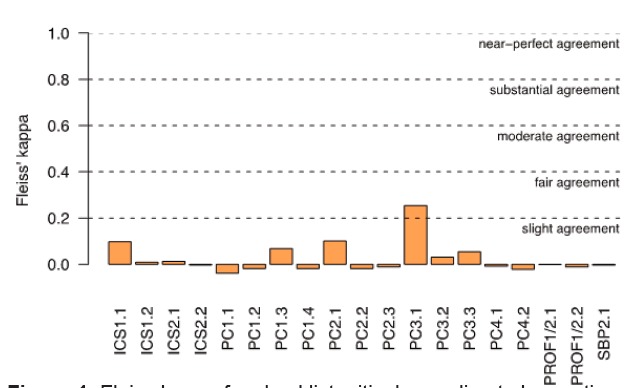
Fleiss kappa for checklist critical care direct observation tool items.

When the videos were scored, all possible responses from the checklist categories were used. The tool utilization demonstrated adequate response process; however, both the Checklist format and Milestone Rating Scale format were found to have very poor inter-rater reliability. In other words, the faculty could not reliably determine the score of each resident despite all viewing the same performance. Fleiss’ kappa of each of the 19 items measured on the Checklist format of the CDOT was near zero for most items and categorized as “slight agreement” for only one item (Figure 1). There was pronounced variability in the raters’ use of the “not applicable” category on the Checklist CDOT format; thus, we also calculated Fleiss’ kappa excluding this category. However, they were found to be just as low—ranging from −0.04 to 0.25.

The Milestone Rating Scale CDOT had a total of nine items. Mean ratings for each item were low, ranging from 2.26 to 2.83 with an acceptable amount of variability (SD ranging from 0.66 to 1.16), and raters used the full range of the scale for most items.

**Figure 2:**
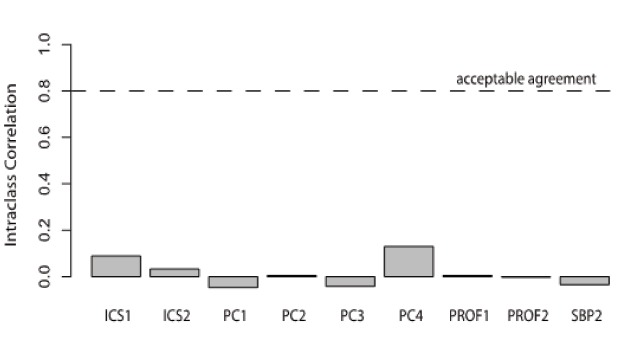
Intra-class correlations for sub-competency milestone level CDOT. *CDOT*, Critical Care Direct Observation Tool

Rater agreement reliability was near zero (Figure 2). The Intra-class correlations (ICC1) statistics were near zero for all items except one (PC4), which had an estimated reliability of 0.13 (see Figure 2). None of these ICC1 statistics approached the acceptable level of inter-rater agreement of ICC1=0.80. This is due largely to the wide range in ratings for each trainee on each item. Each trainee was rated both low (1 or 2) and high (3, 4 or 5) by at least one rater on every item, and trainee mean ratings did not differ by more than one rating point for any item. This pattern was not due to raters’ “hawk/dove” differences. After adjusting ratings so each rater’s mean rating was zero, ICC1s remained low (from −0.04 to 0.019).

## DISCUSSION

In order to adequately implement milestones, educators need objective, reliable assessment tools with data to support the tool’s validity. Although the CDOT was designed with attention to sources of validity evidence, this study found disconcerting results. While each milestone was used essentially verbatim, when used to assess standardized video performance neither the Milestone Checklist nor the Milestone Rating Scale CDOT demonstrated reliability. The reliability analysis demonstrates that essentially raters could not agree on the appropriate scores of the three residents. This is particularly troubling because similar instruments have already been widely adopted by EM programs for resident assessment. Based on the data, the authors do not believe the inter-rater disagreement for both instruments was due to inadequate scale use, range restriction effects, rater bias (hawk/dove), or instrument format.

The underlying issue is error; namely, variance that is not explained by the model. There are two domains of potentially reducible error to consider—rater error and instrument error. This study used the assumption that faculty routinely assess residents on the EM milestones and, for this reason, volunteer faculty raters using direct observation could accurately and consistently judge the performance of videotaped residents. But this clearly did not happen. When analyzing the potential sources of rater error, the following types of bias may be playing a role: rater inconsistency, severity and leniency, frame of reference, central tendency, and the halo effect. Workplace assessments in medicine require judgment on the part of the rater, which suggests that there may be no such thing as a purely objective interpretation of assessment results.^18^

Rater inconsistency in this context occurs when a faculty member fails to apply the rating scale in the same manner as other faculty members. This diminishes the tool’s ability to differentiate between higher achieving and lower achieving residents. 10 Second, leniency and severity biases undermine inter-rater reliability. Leniency bias occurs when a faculty member gives high scores even when a performance is not deserving of such as score (“doves”) and severity bias occurs when raters give low scores despite good performance (“hawks”).^10^–^12^ Third, faculty may tend to use their own clinical practice style as a frame of reference for clinical assessment rather than adhering to the agreed-upon standard. 13 Further, faculty are experts and may take shortcuts in patient care due to expert intuitive judgment that can impact scoring. Fourth, faculty tend to avoid extreme positions on a rating scale, resulting in a central tendency on their assessments, essentially restricting the intended range of the rating scale.^10^ Lastly, when faculty personally know or identify with a particular learner, there may be a tendency to assess that learner more positively (the halo effect).^10^

Intuitively, rater training should minimize these types of rater error. Training assessors how to use assessment tools has been shown to improve reliability in some studies, 14 while having little effect in others. 15 The clinical experience of faculty assessors as well as their knowledge of the intricacies of milestones may affect their trainability. 16 Regardless, the common practice at most residencies is to have faculty without training assess residents on the milestones in the clinical setting.

The second domain of error, instrument error, refers to the difference between the actual skill and the value measured by the rating instrument. When it comes to rating scales, some advocate for making the tool appear less subjective by removing scales (1–5) and inserting yes/no type checklists. In this study, there was no difference between using a checklist compared to a scale. The literature does not definitively advocate for the use of one rating scale over another. 12 Using the EM milestones themselves within the CDOT tool may be another source of instrument error. While the use of milestones phrasing improved content validity, the wording of many of the milestones is relatively broad and may not be useful when used to rate specific behaviors.^17^ One final source of instrument error may be that the number of items is too large, resulting in cognitive overload. Raters may not be able to keep in their working memory all of the items on the scale, resulting in an incorrect score. Unfortunately, there is no true measurement or standard to evaluate how accurately we are judging our residents.

Beeson et al. recently published a study demonstrating that the internal consistency of the subcompetencies using a Cronbach**’**s alpha coefficient was high, although the study did not specifically look at workplace-based assessment and rater variability. This study used the milestone scoring for each resident that was submitted to ACGME. The accompanying editorial noted “there is a need for additional validity evidence from multiple sources, evaluation of potential limiting bias, and defining of the appropriate role of milestones in assessment.

### Limitations

Our study had several limitations. The definition of critical care resuscitation likely varies widely among EM physicians; thus, the context of the case and the idiosyncrasy of faculty judgments are limitations. Faculty training was another limitation. While there was a brief video on rater training, specific attention to helping faculty understand the milestones may have improved the inter-rater reliability. However, modeling after current practice, faculty are routinely rating residents with minimal training. Additionally, the use of videos to collect validity evidence may not represent the actual functioning of the tool in a clinical setting. Finally, the clinical experience of our assessors was not documented. It is possible that the lack of inter-rater reliability could be attributed to a difference in the assessment inferences used by faculty based on the faculty members’ experience.

### Future Directions

What is to be done? First, we continue to advocate for direct observation of workplace-based assessment as a component of milestone evaluation. The use of milestones provides a framework for the very important conversation between faculty and trainee to describe performance and identify areas of excellence and those areas needing improvement. While the CDOT instrument is limited in reliability, it may be effective as a tool to use as a framework for discussion during direct observations of critical patients.

When there are reliability and validity issues, Van der Vleuten argues for the use of programmatic assessment using multiple modalities and lower stakes assessment to achieve a more complete picture of the learner. 18 It is imperative to understand that the milestones are not assessment tools themselves but are constructs against which we reference resident performance. As final milestone assessment for EM residents is a high-stakes summative assessment, the goal of the Clinical Competency Committee should be to incorporate various assessment tools from multiple individuals regarding resident performance in order to make assessments that are as reliable and valid as possible.

## CONCLUSION

Although EM residents are currently being assessed on milestones, the validity and reliability of tools for such assessment have yet to be determined. Implementing milestones-based evaluation is a formidable challenge as we must generate evidence to inform the development of assessment tools. This study was a rigorous attempt to collect validity evidence for an EM milestone direct observation instrument. Despite nearly verbatim use of the EM milestones during construction of this tool, while maintaining content validity, the resulting responses were not reliable and were fraught with variability. This may be secondary to rater- and instrument-based error. However, based on this study, there are significant concerns for the reliability of other EM milestone assessment tools in use that have not been examined in terms of their reliability and validity.

## Figures and Tables

**Table:** Three major soures of test validity.^6^

	Definition	Validity evidence for instrument
Content	The extent to which test content and the construct of interest are matched. Evidence of content validity may include test blueprint to match content to construct, the use of experts in the field, literature and guidelines (e.g., milestones) to determine content match with construct.	1) Using language from the milestones, 2) Involving an expert panel of EM residency leaders from six academic institutions, 3) Using a modified Delphi approach, and 4) Utilizing an assessment blueprint based on a review of each of the EM ACGME sub-competencies and determining the appropriateness of each for incorporation into the direct assessment tool
Response process	The cognitive and physical processes required by the assessment also represent the construct. Decisions for response process validity include: the choice for global score versus checklist; analysis of individual responses; debriefing of respondents; and quality assurance and control of assessment data.	1) Explicit scoring algorithms directly related to the underlying construct, 2) By the judgments of the experts regarding the scoring, 3) Adjustment of scoring responses, 4) Field testing and revision
Internal structure	Assessment content and processes provide data about learner performance relevant to the construct. Internal process refers to how assessment transforms the data into a score that represents the construct. Evidence of internal structure includes: statistical characteristics of items and option functions; factor analysis.	*Reliability of reproducibility of scores *Inter-item correlations

*EM*, emergency medicine; *ACGME*, Accreditation Council for Graduate Medical Education
